# Semaglutide Exacerbates Stunting in Growth-Impaired Juvenile Male Mice via Reduced Energy Metabolism

**DOI:** 10.1210/jendso/bvaf158

**Published:** 2025-10-10

**Authors:** Amélie Joly, Lucas Rebiffé, Yves Dusabyinema, Julien Dellinger, Estelle Caillon, Karine Gauthier, François Leulier, Filipe De Vadder

**Affiliations:** Institut de Génomique Fonctionnelle de Lyon, École Normale Supérieure de Lyon, CNRS UMR 5242, UCBL Lyon-1 Lyon F-69007, France; Institut de Génomique Fonctionnelle de Lyon, École Normale Supérieure de Lyon, CNRS UMR 5242, UCBL Lyon-1 Lyon F-69007, France; Institut de Génomique Fonctionnelle de Lyon, École Normale Supérieure de Lyon, CNRS UMR 5242, UCBL Lyon-1 Lyon F-69007, France; Institut de Génomique Fonctionnelle de Lyon, École Normale Supérieure de Lyon, CNRS UMR 5242, UCBL Lyon-1 Lyon F-69007, France; Institut de Génomique Fonctionnelle de Lyon, École Normale Supérieure de Lyon, CNRS UMR 5242, UCBL Lyon-1 Lyon F-69007, France; Institut de Génomique Fonctionnelle de Lyon, École Normale Supérieure de Lyon, CNRS UMR 5242, UCBL Lyon-1 Lyon F-69007, France; Institut de Génomique Fonctionnelle de Lyon, École Normale Supérieure de Lyon, CNRS UMR 5242, UCBL Lyon-1 Lyon F-69007, France; Institut de Génomique Fonctionnelle de Lyon, École Normale Supérieure de Lyon, CNRS UMR 5242, UCBL Lyon-1 Lyon F-69007, France

**Keywords:** semaglutide, stunting, somatotropic axis, energy metabolism

## Abstract

Animals rely on linear growth to attain their full adult size. The regulators of this multifactorial process, including environmental and endocrine cues, are still incompletely understood. Notably, GLP-1, glucagon-like peptide 1 (GLP-1) has emerged as a potential player in this process. Here, we employ semaglutide, a pharmaceutical GLP-1R agonist as a tool to mechanistically dissect the interplay between GLP-1 receptor activation, energy metabolism, and linear growth during the juvenile period, independent of its clinical applications. Using a juvenile mouse model, we show that chronic semaglutide treatment lowers blood glucose without affecting food intake or weight gain in juveniles with a normal growth pattern. However, in growth-stunted juveniles, semaglutide treatment exacerbates linear growth impairment through at least 2 concomitant mechanisms: a moderate reduction in food intake, and a decreased catabolic activity incompatible with tissue growth. These data suggest a complex interplay between GLP-1 signaling, energy metabolism, and growth during juvenile development. Overall, these findings highlight the value of semaglutide as a mechanistic tool for understanding how GLP-1 receptor activation modulates growth and metabolism in juveniles, emphasizing the importance of developmental context for interpreting its effects.

The juvenile period is characterized by intensive growth, a highly energy-demanding process. In mammals, linear growth is regulated by the somatotropic axis, governed by growth hormone (GH) produced by the pituitary gland that stimulates liver production of insulin-like growth factor 1 (IGF-1). Both GH and IGF-1 instruct peripheral tissues to promote tissue and bone growth [1]. In addition to the GH/IGF-1 pathway, metabolic signals including blood insulin levels or tissue metabolic rate influence linear growth rate. Thus, linear growth is a multifactorial process integrating numerous cues including environmental signals (eg, diet [2, 3]) and intrinsic signals (eg, sex hormones [4, 5]). However, not all regulators of this complex physiological process have been identified.

Glucagon-like peptide 1 (GLP-1) is a small peptide hormone secreted by specialized enteroendocrine cells of the intestinal epithelium. Its secretion is stimulated by dietary and microbial signals [6]. The functions of GLP-1 are pleiotropic, including potentiation of insulin signaling, regulation of food intake, and of tissue metabolic rate [7]. Interestingly, both in vitro and in vivo work in rodents has demonstrated that GLP-1 receptor agonists (GLP-1Ras) can promote growth-related phenotypes by enhancing osteoblast proliferation and activity [8, 9]. In addition, several studies in mice have shown that GLP-1 and GLP-1Ras may enhance IGF-1 receptor signaling pathways [10, 11], favor bone metabolism homeostasis [8, 12], and even directly cross-activate the IGF-1 receptor in human cardiac fibroblasts [13]. In addition, short-term or long-term administration of GLP-1Ras in healthy adult volunteers has been shown to increase circulating GH levels [14]. Thus, it has been suggested that GLP-1R signaling could interact with the somatotropic axis [15] and potentially be a regulator of linear growth.

Despite these insights, how GLP-1 signaling and GLP-1Ras affect growth and energy balance during the juvenile period remains largely unexplored. In this study, we examined the effects of chronic treatment with a GLP-1Ra, semaglutide (Sema), on growth and energy metabolism in nondiabetic juvenile mice under optimal and impaired growth conditions. Importantly, this study is not intended to model pediatric obesity or predict clinical outcomes. Our objective was to explore the physiological role of GLP-1 receptor agonism during juvenile development using a mechanistic mouse model. We report that Sema treatment improves glucose metabolism of nonobese, nondiabetic juvenile mice, irrespective of their growth phenotype. However, while it does not affect growth in normally developing mice, Sema treatment further compromises linear growth in stunted juveniles. Notably, these growth-inhibitory effects appear largely independent of alterations in the somatotropic axis. Besides lowering food intake in stunted mice, gene expression analyses in metabolically active organs including adipose tissues and skeletal muscle suggest that Sema treatment may also impair growth in stunted juveniles by downregulating pathways involved in basal energy expenditure and anabolic metabolism.

## Materials and Methods

### Animals and Treatments

All animal procedures have been approved by a local animal care and use committee (CECCAPP) and subsequently authorized by the French Ministry of Research (APAFIS No. 40481-2022103017295302 v3).

C57BL/6N male mice aged 21 days were purchased from Charles River and weaned onto either the standard AIN-93G diet (control diet, CD) or on an isocaloric, protein-restricted modification of the AIN-93G designed to induce stunting (stunting-inducing diet, SID), as previously described in our study [3]. The detailed composition of both diets is provided in Supplementary Table S1 [16]. Animals were weighed and body size was measured weekly under brief isoflurane anesthesia. They received subcutaneous injections of Sema (Ozempic, Novo Nordisk 0.5 mg injectable solution) at a dose of 30 nmol/kg body weight, or vehicle (Veh; 0.9% NaCl), twice weekly until postnatal day 56 (P56). Two independent cohorts of animals were used for this study. The first cohort included 8 animals per group and the second cohort had 7 animals per group. Injections were administered 1 day prior to the oral glucose tolerance test (OGTT), and 1 day before the animals were humanely killed.

At P56, animals were fasted for 6 hours starting at 8 Am and killed by cervical dislocation at 2 Pm. Blood and organs were sampled. The left femur and tibia were dissected and measured precisely using a caliper.

For OGTT, mice were fasted for 6 hours starting at 8 Am and gavaged with a 20% D-(+)-glucose solution (Sigma Aldrich, G7021) at 1 g/kg body weight. Blood glucose was measured from the tail vein at 0 (baseline), 15, 30, 60, 90, and 120 minutes post gavage using a handheld glucometer (OneTouch Verio Reflect, LifeScan Europe). Blood samples were collected from the tail vein at 0, 15, and 30 minutes, serum was extracted and stored at −20 °C until further processing.

Only male mice were included in this study, as we previously demonstrated that SID induces greater growth impairment in juvenile males compared to females [3].

### Insulin Measurements

Serum insulin levels were measured using the Ultra Sensitive Insulin enzyme-linked immunosorbent assay (ELISA) kit (Crystal Chem catalog No. 90080, RRID:AB_2783626), following the manufacturer's instructions.

### Western blotting

Frozen liver samples were processed for Western blotting as described by Schwarzer et al [17]. Briefly, samples were lysed in radioimmunoprecipitation assay (RIPA) Plus lysis buffer (50 mM Tris, 1% IGEPAL, 0.5% sodium deoxycholate, 0.1% SDS, 150 mM NaCl, 2 mM EDTA, 50 mM NaF, protease inhibitors). Protein concentration was measured using the BCA assay (Thermo Scientific, 23227). Equal amounts of protein (30 µg per well) were separated in Mini-PROTEAN TGX Stain Free precast gels (Bio-Rad, 4868096), and transferred to nitrocellulose membranes using the Trans-Blot Turbo system (Bio-Rad). Membranes were blocked and incubated for 1 hour at room temperature with either rabbit monoclonal anti-total protein kinase B (Akt) (C67E7) (1:1,000, Cell Signaling, Technology catalog No. 4691, RRID:AB_915783), or rabbit monoclonal anti-phospho S473 Akt (D9E) (1:2000, Cell signaling Technology catalog No. 4060, RRID:AB_2315049). After washing, membranes were incubated for 1 hour at room temperature with horseradish peroxidase–conjugated anti-Rabbit immunoglobulin G (IgG) secondary antibody (1:2500, Promega catalog No. W4011, RRID:AB_430833). Detection was performed using ECL Western Blotting Substrate (Promega, W1001), and signals were captured with the Amersham IQ800 imager (Cytiva). Quantification was performed using Image Lab software version 6.1 (Bio-Rad), using total protein normalization [18].

### Insulin-like Growth Factor 1 Quantification

Liver tissues were weighed and homogenized in lysis buffer containing phosphate-buffered saline, 1% Triton × 100, and protease inhibitors (Roche, cOmplete, EDTA-free). Homogenates were centrifuged (12 000*g*, 10 minutes, 4 °C), supernatants were used to quantify IGF-1 levels using the Mouse/Rat IGF-1 Quantikine ELISA (R & D Systems catalog No. MG100, RRID:AB_2827989). Total protein content was measured using the BCA assay for normalization. IGF-1 concentrations are expressed as pg/*μ*g total protein. The same kit was used to quantify circulating IGF-1.

### Gene Expression Analysis

Total RNA was extracted from brown adipose tissue (BAT) and subcutaneous white adipose tissue (scWAT) using TRI Reagent (Invitrogen, M9738). Briefly, tissues were homogenized in TRI reagent, centrifuged to remove debris, and RNA was isolated via chloroform phase separation followed by isopropanol precipitation. For liver and skeletal muscle, total RNA was extracted using the NucleoSpin RNA kit (Macherey-Nagel, 740955.250).

Reverse transcription was performed on 1 *μ*g of RNA using the SensiFAST complementary DNA (cDNA) Synthesis Kit (Meridian Biosciences, BIO-65053). Quantitative polymerase chain reaction (PCR) was performed using Takyon No ROX SYBR Mastermix blue dTTP (Eurogentec, UF-NSMT-B0701) using a Bio-Rad CFX96 apparatus. Analysis followed MIQE (Minimum Information for Publication of Quantitative Real-Time PCR Experiments) guidelines [19]. Amplification efficiency was evaluated for each primer pair using standard curve assays performed prior to experimental runs. All reactions were carried out in technical duplicates to ensure reproducibility. At least 2 validated reference genes were used for normalization, with gene stability confirmed using the geNorm algorithm available within the CFX96 software. Primers sequences for all reference and target genes are listed in Supplementary Table S2 [16].

### Liver RNA Sequencing Analysis

The concentration and quality of 10 DNAse-treated RNA samples (from SID-fed juveniles, 5 Veh-treated, and 5 Sema-treated) were assessed using the Qubit 4.0 fluorometer (Thermo Fisher Scientific) and the TapeStation 4150 system (Agilent Technologies), respectively. For each sample, messenger RNA was purified from 1 µg of total RNA using oligo(dT)-based magnetic bead isolation (Poly(A) messenger RNA (mRNA) Magnetic Isolation Module, New England Biolabs), following the manufacturer's protocol.

Single-indexed libraries were built using the CORALL Total RNA sequencing (RNA-Seq) Library Prep Kit (Lexogen), according to the manufacturer's instructions. This fragmentation-free protocol ensures full-length transcript coverage. Unique molecular identifiers (UMIs; 12-bp tags) were introduced during the linker Ligation step, enabling the identification and removal of PCR duplicates during downstream analysis to reduce amplification bias.

After quality control (Qubit 4.0 and TapeStation) analysis, libraries were pooled together in equimolar concentrations and sequenced on 2 high-output runs in a single-end mode (1 × 86 bp) using a NextSeq500 Illumina sequencer (Illumina). A total of 1.082 billion reads were generated, yielding an average of 36 million reads per sample (range, 31-44 million), after demultiplexing.

Read preprocessing was performed using a custom pipeline derived from Lexogen's recommendations. Briefly, adapter trimming was performed using the Cutadapt package (version 3.5) [20] and read quality was assessed using FastQC. Read mapping was achieved with STAR (Spliced Transcripts Alignment to a Reference, version 2.7.11a) [21] using the *Mus musculus* GRCm39 genome reference (Ensembl, release 110). UMI-based deduplication was carried out using UMI-tools dedup (version 1.1.2) [22], enabling accurate removal of PCR duplicates. Gene-level quantification was performed with htseq-count (version 2.0.3) [23], generating raw count tables for each sample.

Differential analysis was performed in R (v.4.3.1), using the DESeq2 package (v.1.42.0) [24]. Visualization of differential gene expression was performed using the Enhanced Volcano package (v.1.20.0) [25], applying cutoffs of adjusted *P* value (false discovery rate) less than or equal to 0.01 and log_2_ fold change of 1 or greater. One Veh-treated sample was excluded from analysis following unsupervised clustering and quality control, as it appeared to be an outlier.

The RNA-seq dataset has been deposited in the NCBI Gene Expression Omnibus [26] (GEO) under accession number GSE266477 (https://www.ncbi.nlm.nih.gov/geo/query/acc.cgi?acc=GSE266477).

### Statistical Analysis

Statistical analysis was performed using GraphPad Prism v10.2.1 and R v. 4.4.3. For comparisons between 2 groups, unpaired *t* tests were used when data met assumptions of normality and homogeneity of variance; otherwise, nonparametric Mann-Whitney *U* tests were applied.

Longitudinal data—including body weight, linear size, food intake, and glycemia during the OGTT—were analyzed using linear mixed-effects models in R, employing the lme4 (v. 1.1-4), lmerTest (v. 1.1-36), and emmeans (v. 1.11.0) packages. Mouse identification was included as a random effect to account for repeated measures. Two independent experimental batches were used, and batch was included in all models as a fixed effect along with its interactions with treatment and age. Although the fixed effects involving batch were not statistically significant (*P* > .05), model comparison showed that including the full treatment × age × batch interaction significantly improved model fit. Additionally, the variance attributed to batch as a random effect was consistently small. Therefore, to account for possible batch-related structure while minimizing visual redundancy, representative data from one batch are shown in the figures. Linear mixed-effects models results are detailed in Supplementary Table S3 [16].

## Results

### Chronic Semaglutide Treatment Improves Glucose Tolerance Without Altering Growth of Nondiabetic, Nonobese Juvenile Mice

**Figure 1.**
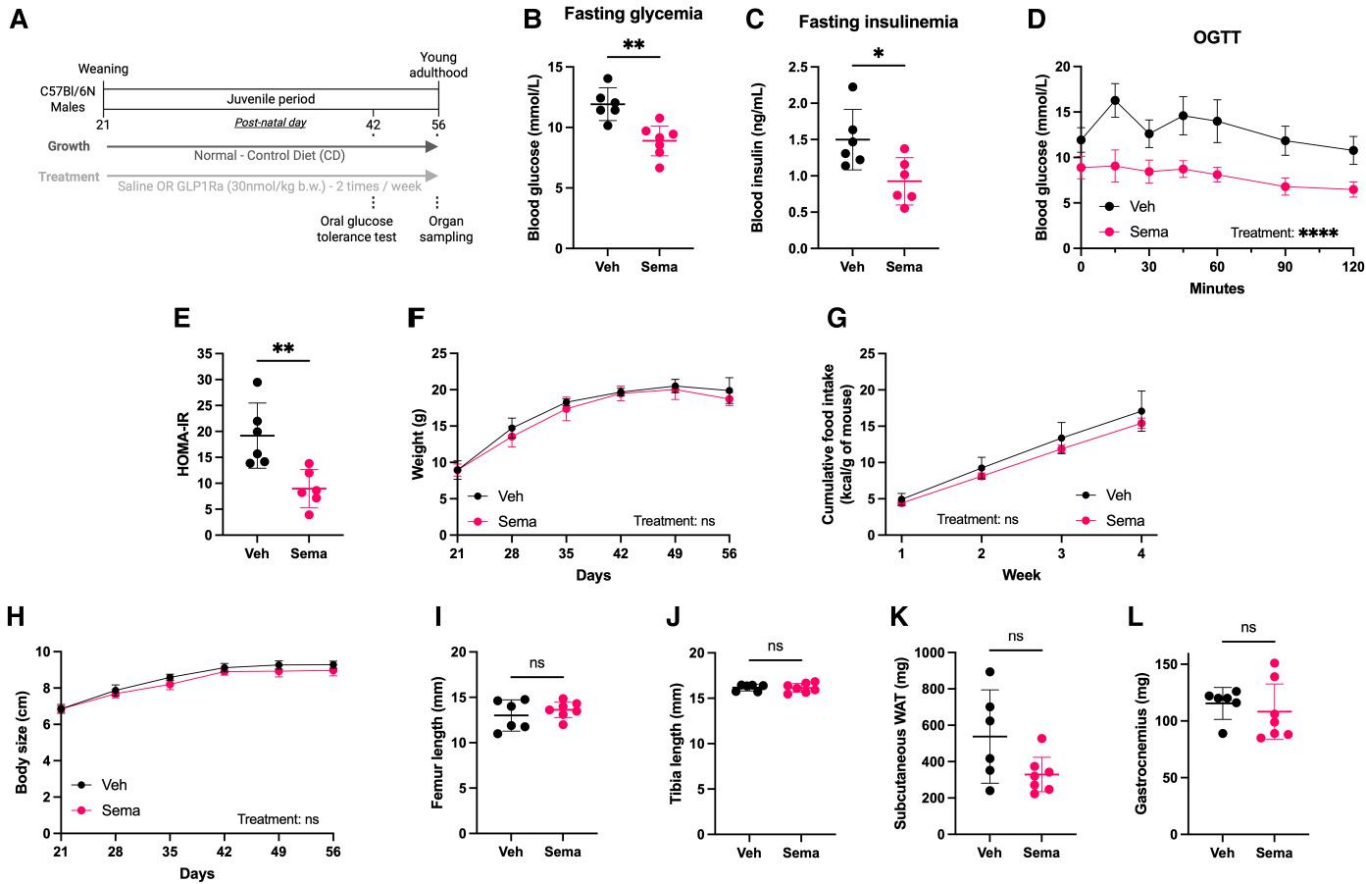
Semaglutide modulates glucose metabolism without affecting growth in control-fed juvenile mice. A, Experimental protocol. Created with Biorender.com. B, Fasting glycemia and C, insulinemia. D, Glycemia during an oral glucose tolerance test (OGTT). E, Homeostatic model assessment of insulin resistance (HOMA-IR). F, Body weight over time. G, Cumulative food intake. H, Body size. I, Femur and J, tibia lengths at postnatal day 56 (P56). Weights of K, subcutaneous white adipose tissue (WAT) and L, gastrocnemius muscle at P56. Sema, semaglutide-treated; Veh, vehicle-treated. Data are shown for 1 batch, representative of both, n = 6-7 per group. For food intake, both batches are represented. Data are shown as mean ± 95% CI. *P less than .05; **P less than .01; ****P less than .0001; ns, not significant.

To determine the effects of chronic Sema treatment during the juvenile developmental window, we first fed weanling male mice an experimental diet meeting the nutritional requirements for optimal growth (AIN-93G [27], hereafter referred to as control diet, CD), and treated with either Veh or Sema (Fig. 1A). Consistent with findings in adult mouse models of obesity [28], chronic Sema treatment lowered fasting glycemia and insulinemia in juvenile mice (Fig. 1B and 1C). Sema treatment also enhanced glucose clearance during an OGTT (Fig. 1D). Interestingly, Sema-treated mice had reduced homeostatic model assessment of insulin resistance (HOMA-IR) (Fig. 1E), which suggests that these mice could have increased insulin sensitivity, a feature that could contribute to the flattened glucose response to OGTT. Surprisingly, in contrast to its known effects in mouse models of diet-induced obesity [29-31], chronic Sema treatment did not reduce body weight or food intake in juvenile animals maintained under optimal nutritional conditions (Fig. 1F and 1G). Linear growth was also unaffected, as shown by similar body size (Fig. 1H) and bone length (femur and tibia; Fig. 1I and 1J) between Veh-treated and Sema-treated mice at the end of the treatment. Assessment of the somatotropic axis revealed no detectable changes in circulating IGF-1 levels or liver *Igf1* and *Ghr* expression (Supplementary Fig. S1 [16]) on Sema treatment, consistent with the absence of growth alterations. Furthermore, Sema treatment did not seem to affect body composition, with similar weights of scWAT and gastrocnemius muscle between groups (Fig. 1K and 1L). Altogether, these results show that chronic Sema treatment appears to modulate glucose metabolism in healthy juvenile mice without affecting growth dynamics.

### Chronic Semaglutide Treatment Exacerbates Growth Impairment in Stunted Juvenile Mice

**Figure 2.**
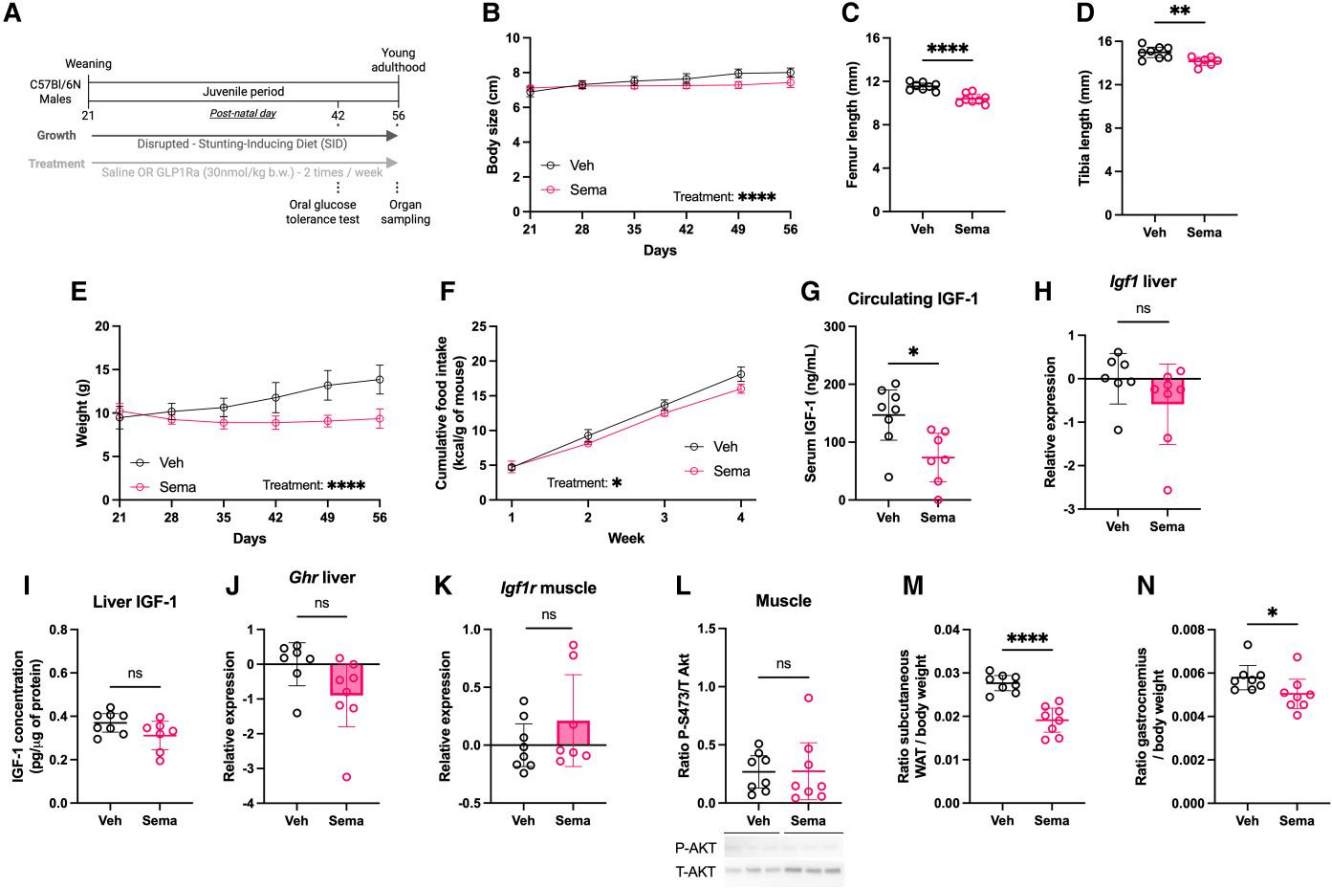
Semaglutide further impairs linear growth and alters body composition in stunted juvenile mice. A, Experimental protocol. Created with Biorender.com. B, Body size. C, Femur and D, tibia lengths at postnatal day 56 (P56). E, Body weight over time. F, Cumulative food intake. Somatotropic axis markers at P56: G, serum insulin-like growth factor 1 (IGF-1) concentration; H, liver *Igf1* expression; I, liver IGF-1 concentration; and J, liver *Ghr* expression. Muscle K, *Igf1r* expression and L, phosphorylated protein kinase B (Akt) (S473) to total Akt ratio measured by Western blot and normalized to total protein content. Relative weights of M, subcutaneous white adipose tissue (WAT) and N, gastrocnemius muscle at P56. Sema, semaglutide-treated; Veh, vehicle-treated. Data are shown for 1 batch, representative of both, n = 7-8 per group. For food intake, both batches are represented. Data are shown as mean ± 95% CI. *P less than .05; **P less than .01; ****P less than .0001; ns, not significant.

Given the importance of linear growth as a hallmark of juvenile physiology and the observed metabolic modulation by Sema under optimal nutritional conditions, we next investigated its effects in a context of disrupted growth (Fig. 2A). Stunting, defined as low height for age [32], is often associated with a lack of dietary protein [33]. Therefore, we modeled stunting in juvenile males by feeding them a low-protein variant of the AIN-93G (referred to as SID; see Supplementary Table S1 [16]) [3]. We have previously demonstrated that this dietary intervention in sufficient to induce major linear growth retardation in juvenile male mice, associated with perturbation in the somatotropic axis function [2, 3]. Unexpectedly, Sema treatment further impaired linear growth in SID-fed juveniles, resulting in a 7% reduction in body size compared to Veh-treated controls, along with corresponding reductions in femur and tibia length (Fig. 2B-2D). These findings suggest that Sema treatment disrupts body growth under a growth-limiting nutritional environment but not under optimal growth conditions, highlighting the context-dependency of its physiological effects.

Sema is known to reduce body weight, largely via appetite suppression, in patients with obesity [29-31] and in adult mouse models [28]. In SID-fed juveniles, Sema reduced weight gain (32% lower than Veh-treated counterparts) and reduced their caloric intake by approximately 17% over the procedure (Fig. 2E and 2F). While this reduction in food intake can contribute to the further impairment of body weight and size growth in SID mice treated with Sema, we decided to explore other potential effects of Sema on the mechanisms governing growth.

Given previous reports of GLP-1Ras interfering with IGF-1 signaling [15], we examined the somatotropic axis in SID-fed mice treated with Sema. Circulating IGF-1 levels were reduced in Sema-treated animals (Fig. 2G), consistent with impaired growth. However, liver expression of *Ghr* and *Igf1*, as well as hepatic IGF-1 protein levels, were unchanged between Sema- and Veh-treated animals (Fig. 2H-2J), arguing against a disruption of GH signaling at the hepatic level. Since IGF-1 promotes anabolic processes in peripheral tissues such as skeletal muscle [34], we further assessed IGF-1 signaling in this tissue. Sema treatment did not affect *Igf1r* expression or downstream AKT phosphorylation in skeletal muscle (Fig. 2K and 2L). Taken together, these results do not support major alterations in somatotropic axis function as the primary mechanism underlying Sema's growth-inhibitory effects in stunted juveniles.

Interestingly, Sema-treated SID-fed mice displayed signs of altered body composition, with lower relative weights of scWAT and gastrocnemius muscle (Fig. 2M and 2N). This prompted us to examine glucose metabolism in these animals. As previously described [3], SID-fed mice had decreased fasting glycemia and insulinemia compared to CD-fed animals (Supplementary Fig. S2A [16]). In addition, Sema improved glycemic response to OGTT and lowered fasting glycemia, similar to effects observed in CD-fed animals (Supplementary Fig. S2A and S2B [16]). However, fasting insulinemia and HOMA-IR were not significantly altered in SID-fed mice (Supplementary Fig. S2C and S2D [16]). Overall, our data show that chronic Sema treatment modulates linear growth, energy metabolism, and body composition in growth-impaired juveniles and is associated with a moderate food intake reduction, independently of changes in insulin levels or major alterations of the somatotropic axis.

### Semaglutide Alters Energy Metabolism-related Gene Expression in Metabolically Active Tissues of Stunted Juvenile Mice

**Figure 3.**
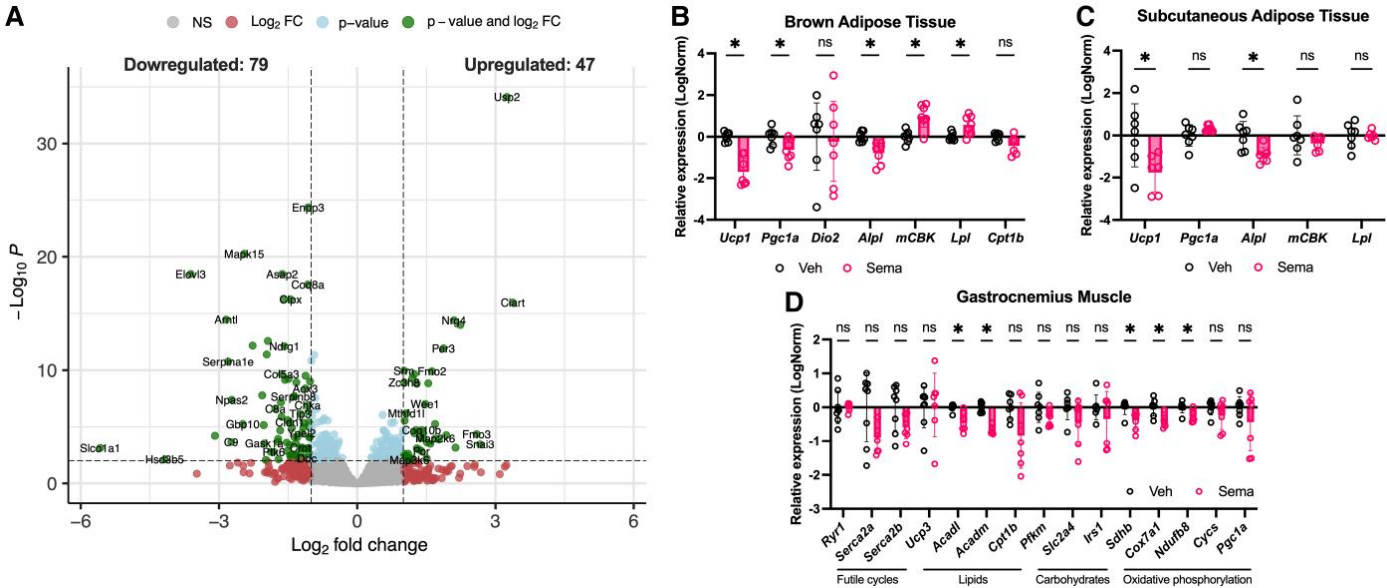
Semaglutide alters energy metabolism gene expression in metabolically active tissues in stunted juvenile mice. A, Volcano plot showing differential gene expression in the liver of stunting-inducing diet–fed juveniles treated with semaglutide (N = 5) vs vehicle (N = 4). Relative expression of energy metabolism-related genes in B, brown adipose tissue (BAT); C, subcutaneous adipose tissue; and D, gastrocnemius muscle. The reference genes used to normalize gene expression are: *Rpl32* and *Rsp18* for BAT and white adipose tissue (WAT); *Gapdh* and *Tbp* for muscle. Sema, semaglutide-treated; Veh, vehicle-treated. Data are shown for 1 batch, representative of both, *n* = 7-8 per group. Data are shown as mean ± 95% CI. *P less than .05; **P less than .01; ****P less than .0001.

To better understand how Sema affects growth-compromised juveniles, we analyzed several metabolically active tissues. The liver is a central organ both for growth and metabolic regulation, and GLP-1 receptors are expressed in specific liver endothelial cells [35]. We thus reasoned that Sema might exert both direct and systemic effects in this tissue. A transcriptomic analysis of the liver revealed that, among approximately 13 000 detected genes, only 126 were differentially expressed between Sema- and Veh-treated animals (47 upregulated, 79 downregulated) with no signature of alterations in metabolic- or growth-related processes, indicating relatively mild hepatic transcriptional reprogramming (Fig. 3A).

Given the limited hepatic response, we next examined Sema's effect on selected genes' expression profiles in metabolically active peripheral tissues whose metabolic activity is known to be modulated by GLP-1Ras [36]: BAT, scWAT, and gastrocnemius muscle. We focused on genes involved in key energy-expending pathways, including fatty acid oxidation (*Cpt1b*, *Lpl*, *Acadm*, and *Acadl*), thermogenesis (*Ucp1*, *Ucp3*, *Pgc1a*, and *Dio2*), mitochondrial electron transport chain (*Sdhb*, *Cox7a1*, *Ndufb8*, and *Cycs)*, carbohydrate metabolism (*Pfkm*, *Slc2a4*, and *Irs1*), and energy-wasting futile cycles (*Alpl*, *mCBK*, *Serca2a*, and *Serca2b*).

In BAT (Fig. 3B), Sema-treated mice showed reduction of thermogenesis-related genes (*Ucp1* and *Pgc1a*), suggesting decreased thermogenic and oxidative activity. Interestingly, *Lpl* expression was slightly increased, possibly reflecting a compensatory enhancement of lipid uptake—despite reduced thermogenic drive. Expression of futile cycle markers was mixed, with decreased *Alpl* and increased *mCBK*, suggesting altered energy-wasting activity, though the direction of change remains unclear. These data point to altered energy expenditure in BAT following Sema treatment in stunted juveniles.

In scWAT (Fig. 3C), *Ucp1* was similarly downregulated, suggesting reduced beiging capacity. In gastrocnemius muscle (Fig. 3D), we observed decreased expression of *Acadl* and *Acadm* (fatty acid oxidation enzymes), along with lower levels of *Sdhb*, *Cox7a1*, and *Ndufb8*, which are markers of mitochondrial oxidative capacity. Collectively, these transcriptional changes evoke that Sema may suppress catabolic and energy-expending pathways along multiple peripheral tissues. Although our data suggest reduced oxidative capacity, in-depth phenotyping (eg, Seahorse assays), would reinforce our gene expression findings.

Catabolic activity is essential during tissue growth to sustain the high-energy requirements of tissue development. Therefore, reduced thermogenic and oxidative capacity in Sema-treated stunted juveniles may contribute to an overall energy-preserving phenotype that would be incompatible with normal tissue growth.

## Discussion

Linear growth is a complex process whose failure can significantly affect health later in life. Many parameters including nutritional deficiencies, endocrine disorders, or chronic illness can affect growth. However, the full range of factors that regulate this complex physiological process has yet to be discovered. In this study, we aimed to examine the role of the GLP-1 signaling pathway in regulating linear growth in mice. To this aim, we treated juvenile mice under optimal or stunted growth condition with the GLP-1Ra Sema.

In juvenile mice raised under nutritionally optimal conditions, Sema treatment enhanced glucose clearance, consistent with effects described in adult rodents and humans [31, 37, 38]. Notably, these metabolic effects were uncoupled from changes in energy balance considerations, as weight gain and, surprisingly, food intake, remained unchanged. The latter observation contradicts previously published studies in obese or overweight mice reporting that Sema treatment yielded a diminution in food intake during the first days of chronic Sema treatment [31, 39]. However, to our knowledge, no study had investigated the effect of Sema on food intake in lean mice, nor in juveniles. Since we measured food intake only weekly, we cannot exclude that a more regular measurement of food intake would reveal variations in food intake pattern between the GLP-1Ra– and Veh-treated groups, especially during the first days after Sema treatment onset. To our knowledge, this is the first study to report a dissociation between Sema's effects on glucose homeostasis and weight gain in juveniles, suggesting age- and diet-specific mechanisms of action [40]. Chronic Sema treatment did not affect linear growth in these animals, consistent with clinical studies in children with obesity treated with the GLP-1Ra Sema or liraglutide, which reported no evidence of altered growth outcomes [41-43].

As linear growth is a key indicator of juvenile health, we explored the potential interaction between GLP-1Ras and the somatotropic growth axis [15] in the context of growth disruption. Using a model of diet-induced stunting, we found that Sema further impaired linear growth of those juveniles, despite maintaining its beneficial effects on glucose management. We acknowledge that our rodent diets were not certified pesticide free by our manufacturer, which is a potential confounder since residues can interact with the somatotropic axis [44]. The possible interplay between low protein intake, Sema treatment, and pesticide exposure warrants consideration and should be addressed in future studies. However, our investigation of the GH/IGF-1 somatotropic axis did not reveal major alterations in hormone production and signaling, challenging our initial hypothesis that Sema's effects on growth might be mediated through disruption of this pathway. Interestingly, contrary to our observations in control diet-fed mice, Sema treatment on SID feeding induced a progressive reduction in cumulative food intake. However, the pattern of food intake alteration appeared different from what has been observed in obese animals, in which Sema treatment induces a strong decrease in food intake (≤ 68%) only during the first 2 weeks of treatment [39, 31].

Sema has been shown to influence many physiological processes regulating energy expenditure [45-47]. Given the important Sema-mediated growth dampening in stunted juveniles, we hypothesize that a moderate reduction in caloric intake may not, on its own, explain reduced body weight and body size. We hypothesized that the observed growth impairment could result from reduced energy availability, compounding decreased energy intake. Transcriptomic and gene expression analyses in metabolically active tissues revealed signs of decreased catabolic activity and suppressed thermogenesis, particularly in BAT and in skeletal muscle. Interestingly, Sema has already been shown to affect muscle metabolism in obese mice [36, 48]. In particular, a recent study has demonstrated that Sema treatment in obese and diabetic mice is associated with increased muscle oxidative phosphorylation [49]. This further emphasizes that GLP-1Ras affect energy metabolism and interorgan physiology differently in stunted juveniles. Taken together, our findings suggest that Sema treatment in stunted juveniles leads to a shift in energy allocation—reducing both energy-intake and energy-expensive processes such as tissue catabolism and thermogenesis, which together participate in impairing growth. A more precise assessment of whole-body energy metabolism, notably by measuring metabolic rate and the respiratory exchange ratio, would help to better understand how GLP-1Ra treatment rewires energy metabolism in stunted juvenile mice.

In conclusion, our study suggests that the GLP-1 signaling pathway might be involved in the regulation of linear growth during juvenile development in mice under growth-limiting nutritional conditions but not under optimal nutritional conditions. Our findings highlight the complexity of this developmental process, and underscore the need for further studies to investigate the integrated mechanisms of action of GLP-1Ras across physiological states and life stages.

## Abbreviations

Akt: protein kinase B
BAT: brown adipose tissue
CD: control diet
GH: growth hormone
GLP-1: glucagon-like peptide 1
GLP-1Ras: GLP-1 receptor agonists
HOMA-IR: homeostatic model assessment of insulin resistance
IGF-1: insulin-like growth factor 1
OGTT: oral glucose tolerance test
P: postnatal day
PCR: polymerase chain reaction
RNA-Seq: RNA sequencing
scWAT: subcutaneous white adipose tissue
Sema: semaglutide
SID: stunting-inducing diet
UMI: unique molecular identifier
Veh: vehicle
